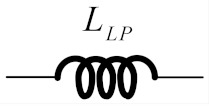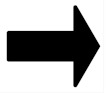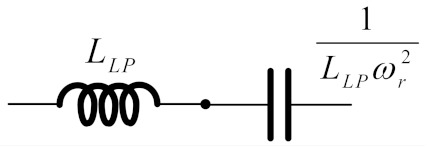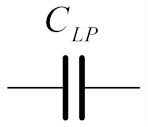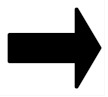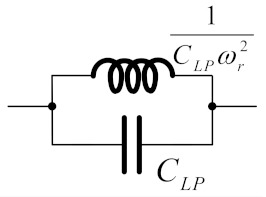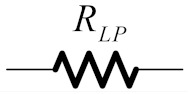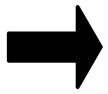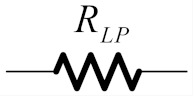# Low-Voltage Low-Pass and Band-Pass Elliptic Filters Based on Log-Domain Approach Suitable for Biosensors

**DOI:** 10.3390/s19245581

**Published:** 2019-12-17

**Authors:** Pipat Prommee, Natapong Wongprommoon, Montree Kumngern, Winai Jaikla

**Affiliations:** 1 Department of Telecommunications Engineering, Faculty of Engineering, King Mongkut’s Institute of Technology Ladkrabang, Bangkok 10520, Thailand; montree.ku@kmitl.ac.th; 2 Department of Electrical Engineering, Faculty of Engineering and Industrial Technology, Silpakorn University, Nakhon Pathom 73000, Thailand; wongprommoon_n@su.ac.th; 3 Department of Engineering Education, Faculty of Industrial Education, King Mongkut’s Institute of Technology Ladkrabang, Bangkok 10520, Thailand; winai.ja@kmitl.ac.th

**Keywords:** analog filter, elliptic, log-domain, low-voltage, tunable, biosensor

## Abstract

This research proposes bipolar junction transistor (BJT)-based log-domain high-order elliptic ladder low-pass (LPF) and band-pass filters (BPF) using a lossless differentiator and lossless and lossy integrators. The log-domain lossless differentiator was realized by using seven BJTs and one grounded capacitor, the lossy integrator using five BJTs and one grounded capacitor, and the lossless integrator using seven BJTs and one grounded capacitor. The simplified signal flow graph (SFG) of the elliptic ladder LPF consisted of two lossy integrators, one lossless integrator, and one lossless differentiator, while that of the elliptic ladder BPF contained two lossy integrators, five lossless integrators, and one lossless differentiator. Log-domain cells were directly incorporated into the simplified SFGs. Simulations were carried out using PSpice with transistor array HFA3127. The proposed filters are operable in a low-voltage environment and are suitable for mobile equipment and further integration. The log-domain principle enables the frequency responses of the filters to be electronically tunable between 10k Hz–10 MHz. The proposed filters are applicable for low-frequency biosensors by reconfiguring certain capacitors. The filters can efficiently remove low-frequency noise and random noise in the electrocardiogram (ECG) signal.

## 1. Introduction

Continuous time filters are essential in electronic sensors and communication devices to remove noise in the circuits. In generic applications, low-order filters are commonplace. First-order filters were used with active devices but were subject to a number of limitations [1,2]. Second-order filters could achieve higher performance but are less ideal for electronic sensors and telecommunications applications due to unsatisfactory cut-off frequency [3].

Further improvements have been made to second-order filters using active building blocks (ABB), such as differential difference amplifier (DDA) [4], voltage differencing transconductance amplifiers (VDTAs) and variable gain amplifiers (VGA) [5], and operational transconductance amplifiers (OTAs) and adjustable current amplifiers (ACAs) [6]. However, they still suffer from limitations inherent in ABBs, including excessive transistors, high-supply voltage, and low-frequency operation. 

In telecommunications, the frequency selective property is essential for modulation and demodulation subsystems. High-order low-pass filters (LPF) and band-pass filters (BPF) play an important role in achieving an accurate frequency and rejecting unwanted frequency spectrum. In [7,8,9,10], higher-order ladder filters were realized using active building blocks, but the filters required floating resistors and capacitors and were less ideal for high frequency operation.

In instrumentation and sensors, very low signals from the sensors, especially in biosensors, are always present and prone to interference [11]. The noisy signals in an electrocardiogram (ECG) and electromyography (EMG) need to be removed to obtain accurate (noiseless) bio-signals which are essential for diagnosis of patients’ health. Besides, in wearable battery-operated biosensor devices, low-supply voltage is imperative to realize compact biosensors with extended battery life.

In 1979, Adams [12] developed continuous time filters (i.e., log-domain filters) which were operable at high frequency and required low power consumption. The log-domain filters were further improved to incorporate many complex structures [13]. In 2008, Psychalinos [14] proposed the log-domain cells containing a large number of transistors. In [15,16], high-performance and low-component-count log-domain cells were incorporated into low-order filters.

In [17], high-order log-domain Chebyshev filters were realized and deployed as LPF and BPF with tunability features. In addition, high-order log-domain elliptic filters were proposed but the performance was unsatisfactory due to the presence of floating capacitors [18], accurate current gain requirement [19], and complicated structure [20]. Complementary Metal Oxide Semiconductor (CMOS)-based high-order elliptic LPF [21] and BPF [22] operable in high frequency were presented, but the accurate current gain requirement posed a significant design challenge.

To overcome the drawbacks inherent in conventional complex high-order elliptic filters, this research proposes simple-structure high-order elliptic LPF and BPF based on log-domain principle. The proposed log-domain elliptic LPF and BPF are realized using differentiators and integrators without accurate current gains and floating passive elements. In addition, the log-domain elliptic LPF and BPF are of wide-range tunability, simple structure, wide dynamic range, and require low power supply.

## 2. Theory and Principle

### 2.1. Analysis of Elliptic Ladder Low-Pass Filter (LPF)

**Figure 1:**
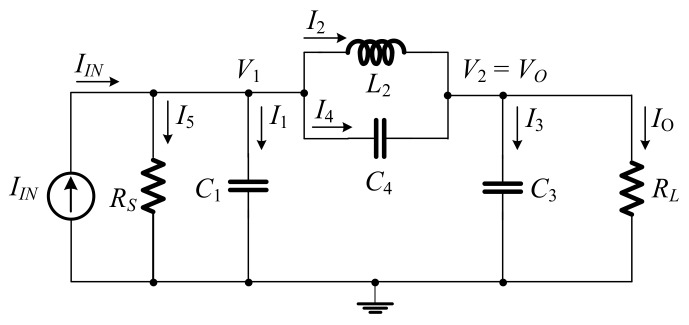
RLC elliptic ladder low-pass filter (LPF) prototype.

Figure 1 illustrates the prototype of Resistor (R), Inductor (L), and Capacitor (C) elliptic ladder LPF [23]. Applying Kirchhoff’s current law (KCL) to Figure 1, the relationships between current (*I*) and voltage (*V*) of LPF can be expressed in Equations (1)–(7).

(1) $$I_{1}=I_{IN}-V_{1}/R_{S}-I_{2}-I_{4},$$

(2) $$I_{2}=\frac{\left(V_{1}-V_{2}\right)}{sL_{2}},$$

(3) $$I_{4}=\left(V_{1}-V_{2}\right)sC_{4},$$

(4) $$V_{1}=\frac{I_{1}}{sC_{1}},$$

(5) $$V_{2}=\frac{I_{3}}{sC_{3}},$$

(6) $$I_{3}=I_{2}+I_{4}-I_{O},$$

(7) $$I_{O}=\frac{V_{2}}{R_{L}}.$$

**Figure 2:**
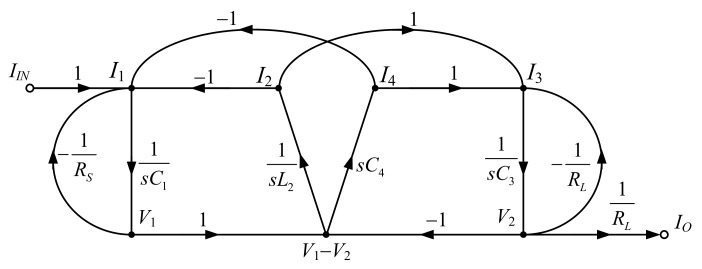
Signal flow graph of the RLC elliptic ladder LPF as shown in Figure 1.

Using the branch currents and nod voltages in Equations (1)–(7), the signal flow graph (SFG) of the RLC elliptic ladder LPF is depicted in Figure 2.

### 2.2. Analysis of Elliptic Ladder Band-Pass Filter (BPF)

**Figure 3:**
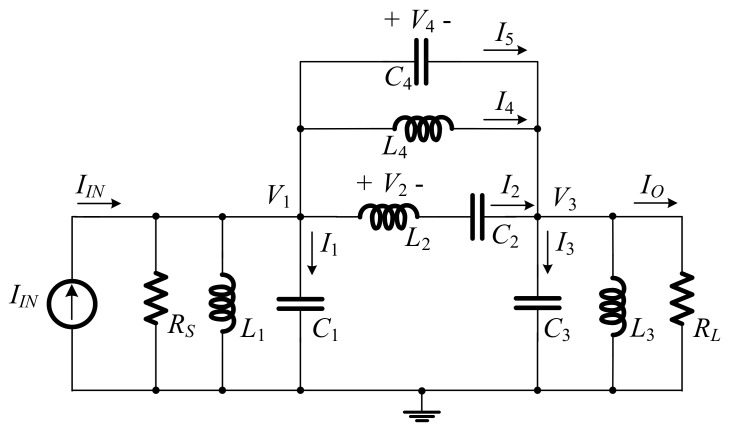
RLC elliptic ladder band-pass filter (BPF) prototype.

To realize BPF from LPF, RLC network transformation methods [23] in Table 1 were applied to the RLC elliptic ladder LPF prototype (Figure 1) and presented in Figure 3.

By applying Kirchhoff’s current law (KCL) to Figure 3, the relationships between current (*I*) and voltage (*V*) of BPF can be expressed as:

(8) $$I_{1}=I_{IN}-I_{2}-(I_{4}+I_{5})-\frac{V_{1}}{sL_{1}}-\frac{V_{1}}{R_{S}},$$

(9) $$I_{2}=\frac{V_{2}}{sL_{2}},$$

(10) $$V_{2}=(V_{1}-V_{3}-\frac{I_{2}}{sC_{2}}),$$

(11) $$I_{3}=I_{2}+(I_{4}+I_{5})-\frac{V_{3}}{sL_{3}}-\frac{V_{3}}{R_{L}},$$

(12) $$I_{4}=\frac{\left(V_{1}-V_{3}\right)}{sL_{4}},$$

(13) $$I_{5}=(V_{1}-V_{3})sC_{4},$$

**Figure 4:**
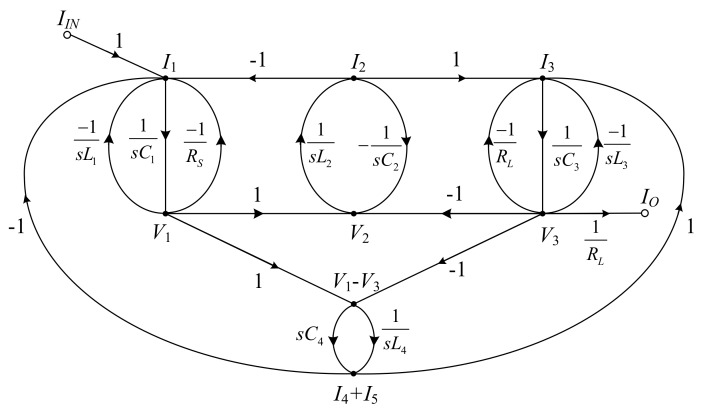
Signal flow graph of the RLC elliptic ladder BPF.

Using the branch currents and node voltages in Equations (8)–(13), the SFG of the RLC elliptic ladder BPF is illustrated in Figure 4.

### 2.3. Log-Domain Lossless Differentiator

**Figure 5:**
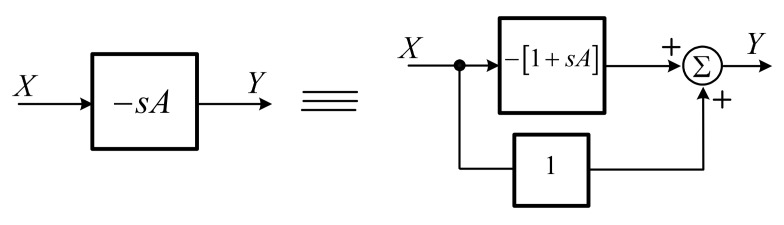
Lossless differentiator realization.

The proposed differentiator is of lossless type with inductor impedance characteristic. A differentiator is generally realized by using an operational amplifier (OPAMP), resistor-capacitor (RC) circuit, or any active building blocks connected with passive elements. Since the proposed lossless differentiator is without feedback [24], it can simply be realized by summation of feedforward of unity-gain with a surplus differentiator (Figure 5) and the transfer function is expressed in Equation (14).

(14) $$\frac{Y}{X}=-sA,$$

**Figure 6:**
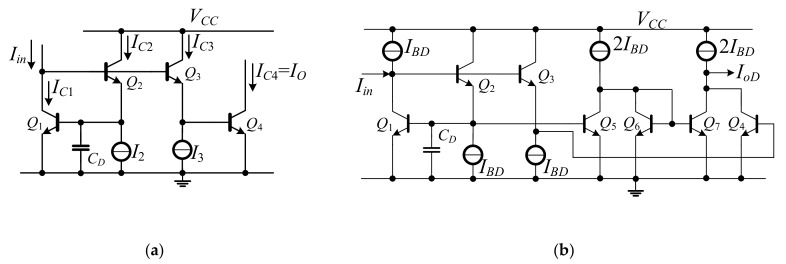
(**a**) Basic log-domain cell, (**b**) complete circuit of log-domain lossless differentiator.

From the block diagram in Figure 5, the log-domain lossless differentiator can be explained by the basic log-domain cell in Figure 6a.

Given *I_C_*_1_ = *I_in_*, *I_C_*_3_ = *I*_3_ and *I_C_*_4_ = *I_O_*, the collector current of *Q*_2_ can be written as:

(15) $$I_{C2}=I_{2}+C_{D}\overset{•}{V_{CD}},$$

The voltage across the capacitor *C_D_* in derivative form is:

(16) $$\overset{•}{V_{CD}}=\frac{dV_{CD}}{dt}=\overset{•}{V_{be1}}=\frac{V_{T}}{I_{in}}\frac{dI_{in}}{dt}=\frac{\overset{•}{I_{in}}V_{T}}{I_{in}},$$

Rewriting (15) and (16) by using translinear principle as:

(17) $$I_{in}\left(I_{2}+\frac{C_{D}\overset{•}{I_{in}}V_{T}}{I_{in}}\right)=I_{3}I_{O},$$

Given the currents *I*_2_ = *I*_3_ = *I_BD_*, then (17) can be rewritten as:

(18) $$\left(I_{in}+\frac{C_{D}\overset{•}{I_{in}}V_{T}}{I_{BD}}\right)=I_{O},$$

Using Laplace transform in (18), the transfer function of surplus lossless differentiator is expressed by:

(19) $$\frac{I_{O}\left(s\right)}{I_{in}\left(s\right)}=\left[1+\frac{sC_{D}V_{T}}{I_{BD}}\right],$$

Equation (19) corresponds to the surplus lossless differentiator function. To transform the surplus lossless differentiator into the inverting type, the input and bias currents (*I*_C1_ = *I_BD_* + *I_in_*, *I*_2_ = *I_BD_*, and *I*_3_ = *I_BD_*) are incorporated as shown in Figure 6b. The transistors (*Q*_5_–*Q*_7_) are included to cancel out the unity term. The final inverting log-domain lossless differentiator function is expressed in (20).

(20) $$\frac{I_{OD}}{I_{in}}=-\frac{sC_{D}V_{T}}{I_{BD}},$$

### 2.4. Log-Domain Integrator

**Figure 7:**
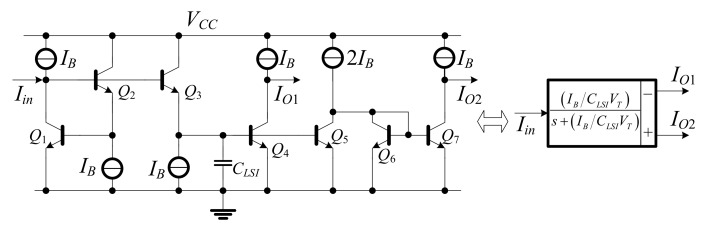
Log-domain lossy integrator and its block diagram.

This section discusses two types of integrators: lossy and lossless integrators. Figure 7 shows the log-domain cell [17] of lossy integrator and can be expressed in the form of negative transfer function in Equation (21).

(21) $$\frac{I_{O1}\left(s\right)}{I_{in}(s)}=\frac{-I_{O2}\left(s\right)}{I_{in}(s)}=\frac{-\left(I_{B}/C_{LSI}V_{T}\right)}{s+\left(I_{B}/C_{LSI}V_{T}\right)},$$

**Figure 8:**
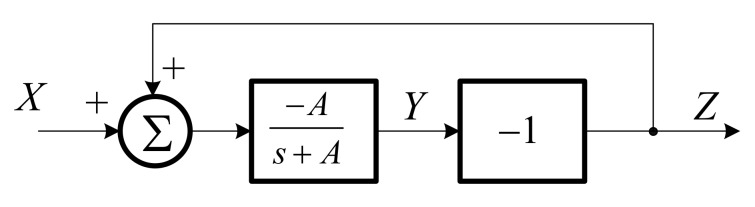
Block diagram of lossless integrator.

The lossless integrator can be obtained by looping back the positive output of lossy integrator to its input. Figure 8 shows the block diagram of lossless integrator by using the lossy integrator.

**Figure 9:**
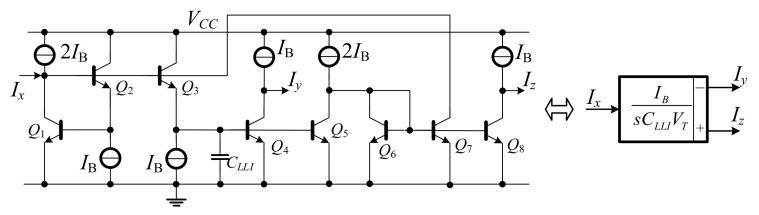
Log-domain lossless integrator.

The log-domain lossless integrator is realized by using loop-back of lossy integrator [17] as shown in Figure 9. The inverting and non-inverting outputs can simply be obtained by *Q*_4_ and *Q*_8_, and the transfer functions are expressed in Equation (22).

(22) $$\frac{I_{y}\left(s\right)}{I_{x}\left(s\right)}=\frac{-I_{z}\left(s\right)}{I_{x}\left(s\right)}=\frac{-I_{B}}{sC_{LLI}V_{T}},$$

## 3. Design of Elliptic Filters

### 3.1. Design of Elliptic LPF

**Figure 10:**
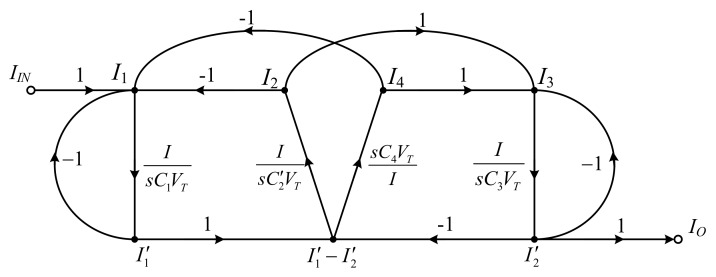
Normalized signal flow graph (SFG) of elliptic ladder LPF.

In Figure 2, the SFG of elliptic ladder LPF can be rewritten in current mode. All voltage variables are converted into current variables using normalized conductance (*I*/*V_T_*). The normalized SFG is depicted in Figure 10.

**Figure 11:**
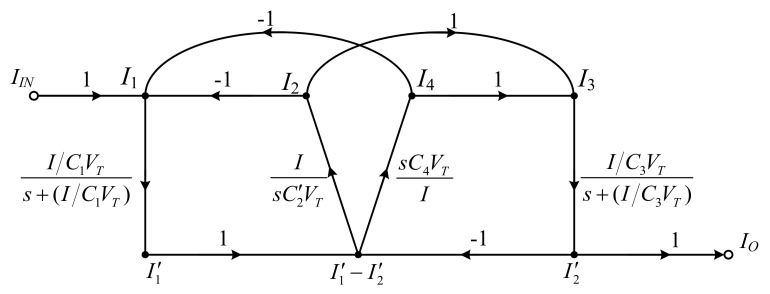
Simplified SFG of elliptic ladder LPF.

Assuming that the terminating resistors at the beginning and end of SFG (*R_S_*, *R_L_*) and normalized resistance (*V_T_*/*I*) are equal, the normalized SFG can be simplified to Figure 11.

**Figure 12:**
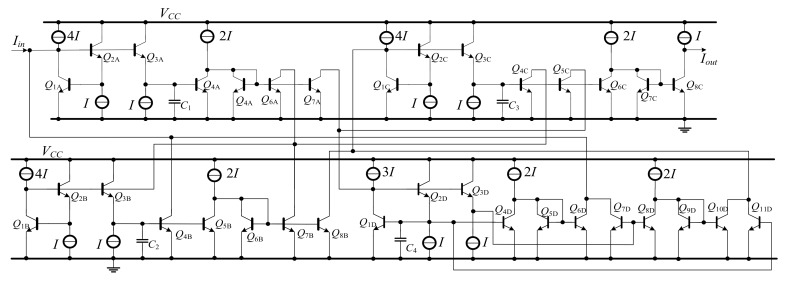
The proposed log-domain elliptic ladder LPF.

The simplified SFG of the elliptic ladder LPF consists of two lossy integrators, one lossless integrator, and one lossless differentiator. In Figure 11, the SFG is much less complex than in [20] and requires no accurate current gain [19,21,22]. Several functions in the simplified SFG (Figure 11) can be readily substituted by log-domain cells. The complete log-domain elliptic ladder LPF is illustrated in Figure 12.

### 3.2. Design of Elliptic BPF

**Figure 13:**
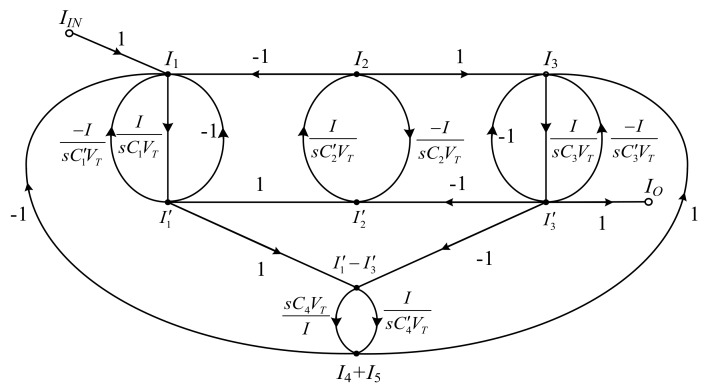
Normalized SFG of BPF.

In Figure 4, the SFG of the elliptic ladder BPF can be rewritten in current mode. All voltage variables are converted into current variables using normalization conductance (*I/V_T_*). The normalized SFG is depicted in Figure 13.

**Figure 14:**
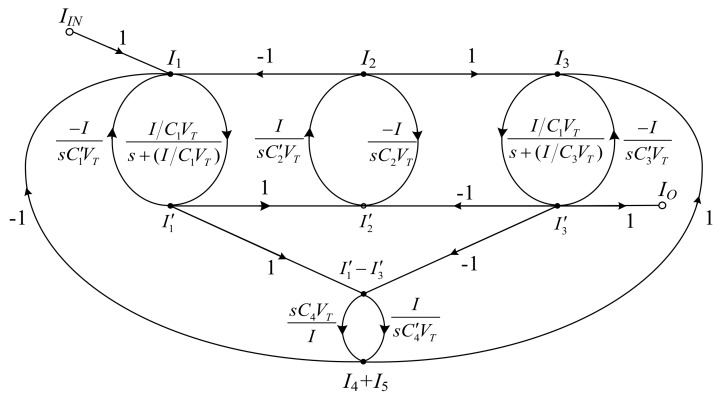
Simplified SFG of BPF.

Assuming that the terminating resistors at the beginning and end of SFG (*R_S_*, *R_L_*) of lossy integrator and normalized resistance (*V_T_/I*) are equal, the normalized SFG can be simplified to Figure 14.

**Figure 15:**
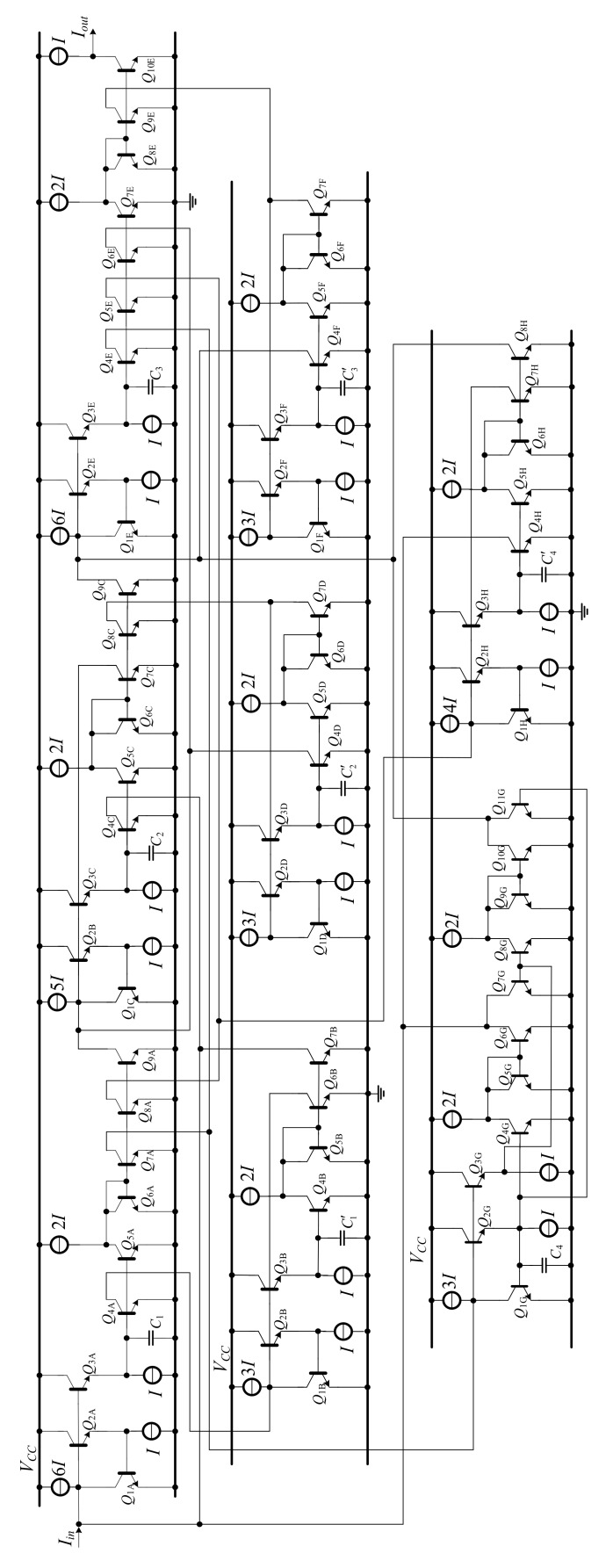
The proposed log-domain elliptic ladder BPF.

The simplified SFG of the elliptic ladder BPF contains two lossy integrators, five lossless integrators, and one lossless differentiator. As seen in Figure 14, the SFG is less complex than that in [20] and requires no accurate current gain [19,21,22]. Many functions in the simplified SFG can be readily substituted by log-domain cells. The complete log-domain elliptic ladder BPF is demonstrated in Figure 15.

## 4. Non-Ideal Analysis

**Figure 16:**
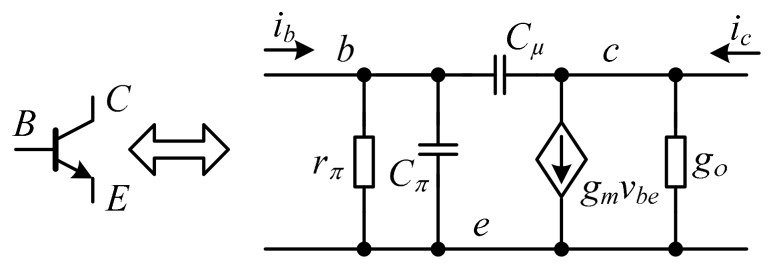
The small signal model of bipolar junction transistor (BJT).

In previous sections, the log-domain cells were analyzed under ideal assumptions. In practice, the cells are influenced by parasitic elements inside bipolar junction transistors (BJT). As a result, the proposed filters are subject to inherent parasitic effects. However, the analysis of parasitic impacts on the filters are highly complicated. The small signal model of BJT is typically used to investigate log-domain cells, as shown in Figure 16. The BJT small-signal model consists of transconductance $\left(g_{m}=I_{C}/V_{T}\right)$, base-emitter parasitic resistance (*r_π_*), parasitic conductance of the collector-emitter $\left(g_{O}=I_{C}/\left(V_{A}+V_{CE}\right)\approx I_{C}/V_{A}\right)$, parasitic base-emitter capacitance (*C_π_*), and parasitic base-collector capacitance (*C_µ_*). For ease of investigation, the transconductance (*g_m_*) and parasitic capacitances (*C_π_* and *C_µ_*) of all transistors are assumed to be identical.

### 4.1. Non-Ideality Effects of Parasitic Resistance (r_π_), Conductance (g_o_) and Finite Beta (β)

Finite beta (*β*) contributes to gain error in the low frequency range [12]. Meanwhile, collector-emitter parasitic conductance ($g_{O}=I_{C}/\left(V_{A}+V_{CE}\right)\approx I_{C}/V_{A}$), which is inversely proportional to the early voltage (*V_A_*), and base-emitter parasitic resistance ($r_{\pi }=\beta /g_{m}$) collectively influence the response accuracy of a translinear circuit in the high-frequency range. In Figure 16, the effects of *r_π_* and *g_o_* on the differentiator transfer function, neglecting the parasitic capacitances (*C_π_* and *C_µ_*), can be expressed in Equations (23) and (24), respectively.

(23) $$\frac{I_{ODn1}}{I_{in}}\approx -\left[\frac{C_{d}}{g_{m}}\left(\frac{s\beta g_{m}/C_{d}}{s+\beta g_{m}/C_{d}}\right)+\frac{1}{\beta }\left(\frac{\beta g_{m}/C_{d}}{s+\beta g_{m}/C_{d}}\right)\right],$$

(24) $$\frac{I_{ODn2}}{I_{in}}\approx -\frac{C_{d}}{g_{m}}\left(\frac{sg_{m}^{2}/g_{o}C_{d}}{s+g_{m}^{2}/g_{o}C_{d}}\right),$$

In Equations (23) and (24), the differentiator transfer functions are applicable in the low- to certain high-frequency range. In Equation (23), the differentiator is directly affected by *β* and functions as a high-gain high-pass filter along the applicable frequency range; and as an LPF at the cut-off frequency. In Equation (24), the differentiator is influenced by conductance (*g_o_*), as evident in the high-frequency pole at high frequency beyond the operational frequency.

Similarly, the effects of *r_π_* and *g_o_* on the transfer function of the lossless integrator, neglecting parasitic capacitances (*C_π_* and *C_µ_*), can respectively be expressed in Equations (25) and (26).

(25) $$\frac{I_{OLLIn1}}{I_{in}}\approx -\frac{\beta^{2}}{2}\left(\frac{2/g_{m}r_{\pi }^{2}C_{1}}{s+2/g_{m}r_{\pi }^{2}C_{1}}\right),$$

(26) $$\frac{I_{OLLIn2}}{I_{in}}\approx -\frac{g_{m}}{g_{o}}\left(\frac{g_{o}/C_{1}}{s+g_{o}/C_{1}}\right),$$

In Equations (25) and (26), the integrator is directly affected by base-emitter parasitic resistance (*r_π_*) and conductance (*g_o_*). The integrator functions as an LPF with pole and high gain at low frequency. Indeed, the effect of *g_o_* on the integrator transfer function is greater than *r_π_*.

For the log-domain lossy integrator, the effect of finite beta (*β*) on lossy integrator transfer function and its pole frequency (Figure 7) can be rewritten as Equations (27) and (28), where *I_OLSIn_* and *I_in_* denote the specific cases of small signal analysis.

(27) $$\frac{I_{OLSIn}}{I_{in}}\approx \frac{-\beta \left(\beta^{2}+3\beta \right)}{\beta \left(\beta^{2}+3\beta \right)+sr_{\pi }C_{1}\left(\beta^{2}+2\beta \right)},$$

(28) $$\omega_{LSIn1}\approx \frac{g_{m}\left(\beta^{2}+3\beta \right)}{C_{1}\left(\beta^{2}+2\beta \right)},$$

In Equation (28), *β* is higher than 100 and has very small impact on the frequency response of lossy integrator. The effect of output conductance (*g_o_*) on log-domain lossy integrator can be investigated by the transfer function (Equation (29)) and its pole frequency (Equation (30)).

(29) $$\frac{I_{OLSIn}}{I_{in}}\approx \frac{-(g_{m}^{3}+2g_{m}^{2}g_{o})}{\left(g_{m}^{3}+2g_{m}^{2}g_{o})+sC_{1}(g_{m}g_{o}+g_{m}^{2}\right)},$$

(30) $$\omega_{LSIn1}\approx \frac{g_{m}(g_{m}+2g_{o})}{C_{1}(g_{m}+g_{o})},$$

In Equation (30), the output conductance (*g_o_*) is generally very small compared to its transconductance (*g_m_*) (i.e., *g_o_* << *g_m_*). The output conductance has very small impact on the frequency response of lossy integrator.

### 4.2. Non-Ideality Effects of Parasitic Capacitance (C_π_ and C_µ_)

Parasitic capacitances (*C_π_* and *C_µ_*) affect response accuracy and gain error in the high frequency range. Given Figure 16 and neglecting parasitic resistance (*r_π_*) and conductance (*g_o_*), the effects of *C_π_* and *C_µ_* on the differentiator transfer function can respectively be approximated by Equations (31) and (32).

(31) $$\frac{I_{ODn3}}{I_{in}}\approx \frac{-s\left(C_{\pi }+C_{D}\right)/3C_{\pi }}{s+g_{m}/3C_{\pi }},$$

(32) $$\frac{I_{ODn4}}{I_{in}}\approx \frac{-s(C_{D}/4C_{\mu })}{s+g_{m}/4C_{\mu }},$$

The parasitic capacitors *C_π_* and *C_µ_* also affect the lossless integrator transfer function, and their respective transfer functions are expressed in Equations (33) and (34).

(33) $$\frac{I_{OLLI3}}{I_{in}}\approx \frac{-g_{m}}{s\left(C_{LLI}+C_{\pi }\right)},$$

(34) $$\frac{I_{OLLI4}}{I_{in}}\approx \frac{-g_{m}}{s\left(C_{LLI}+5C_{\mu }\right)},$$

Similarly, *C_π_* and *C_µ_* also affect the lossy integrator transfer function, and the transfer functions are respectively expressed in Equations (35) and (36).

(35) $$\frac{I_{OLSI3}}{I_{in}}\approx \frac{-g_{m}}{g_{m}+s(3C_{\pi }+C_{LSI})},$$

(36) $$\frac{I_{OLSI4}}{I_{in}}\approx \frac{-g_{m}}{g_{m}+s(4C_{\mu }+C_{LSI})},$$

In Equations (31)–(36), the natural frequencies of the differentiator and both integrators are minimally affected by parasitic capacitances (*C_π_* and *C_µ_*). The natural frequencies of the differentiator and the integrators are in the form of $\omega_{d}=I/C_{d}V_{T}=g_{m}/C_{d}$ and $\omega_{i}=I/C_{i}V_{T}=g_{m}/C_{i}$. For the differentiator and the integrators to be operable in high frequency range, either bias current is increased or capacitor value is decreased. For low-power utilization, the bias current must be minimized while the capacitor value is increased. To mitigate natural frequency error, the capacitors *C_d_* and *C_i_* should be:

(37) $$C_{d}>>C_{\pi },\;C_{LLI}>>C_{\pi }+5C_{\mu }\;\mathrm{and}\;C_{LSI}>>C_{3\pi }+4C_{\mu },$$

The 2nd- and 3rd-order poles are also included in Equations (31)–(36), which are beyond the operational frequency of the differentiator and the integrators. These high-order poles can, therefore, be neglected.

## 5. Simulation Results

To verify the performance of the proposed elliptic LP and BP filters, PSpice simulation was carried out. To minimize errors as per the nonideal analysis, the capacitors in the circuits should be 50 pF or higher. The supply voltage was as low as 1.5 V (2*V_BE_*_on_ + *V_comp_*), which was adequate for compressed voltage swing (*V_comp_*) around 0.3 V. The HFA3127 transistor array was used in the simulation [15].

**Figure 17:**
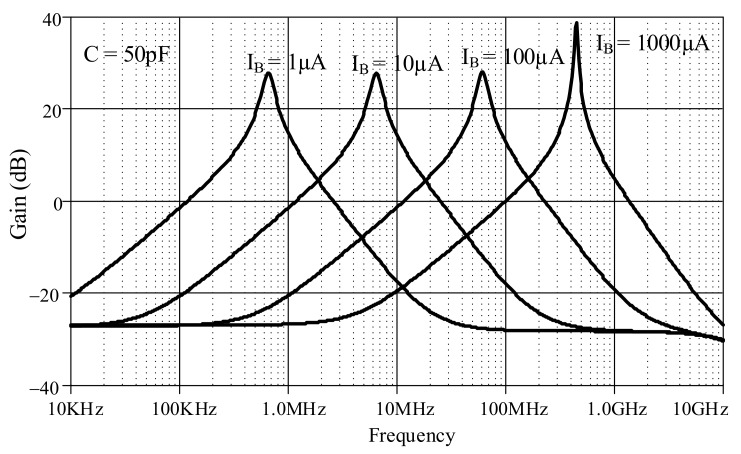
Magnitude responses of lossless differentiator under varying I_B_.

**Figure 18:**
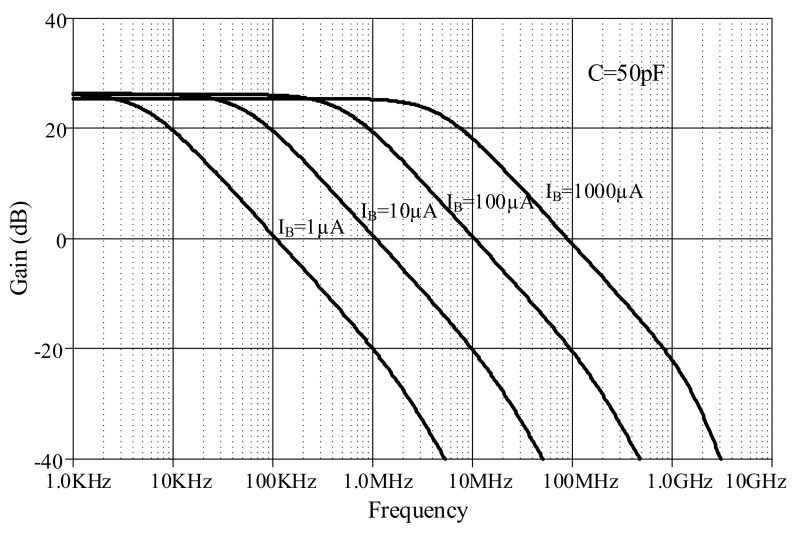
Magnitude responses of lossless integrator under varying *I_B_*.

**Figure 19:**
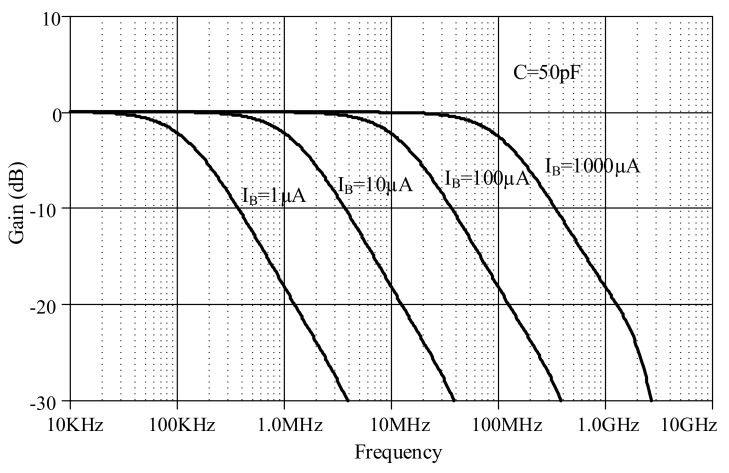
Magnitude responses of lossy integrator under varying *I_B_*.

The proposed lossless differentiator was simulated by varying *I_B_* between 1 µA, 10 µA, 100 µA, and 1000 µA with C = 50 pF, as shown in Figure 17. The magnitude responses varied in response to varying *I_B_*. The differentiator is directly affected by finite *β* as low-pass function at high-frequency which explained in equation (23). Note that the effect of finite *β* is far from the operating frequency of differentiator. Figure 18 shows the magnitude response of the lossless integrator under variable *I_B_* (i.e., 1 µA, 10 µA, 100 µA, and 1000 µA) with C = 50 pF. Figure 19 illustrates the magnitude response of the lossy integrator or first-order LPF under varying *I_B_* (1 µA, 10 µA, 100 µA, and 1000 µA) with C = 50 pF. The simulation results of the differentiator and integrators are in good agreement with theory.

**Figure 20:**
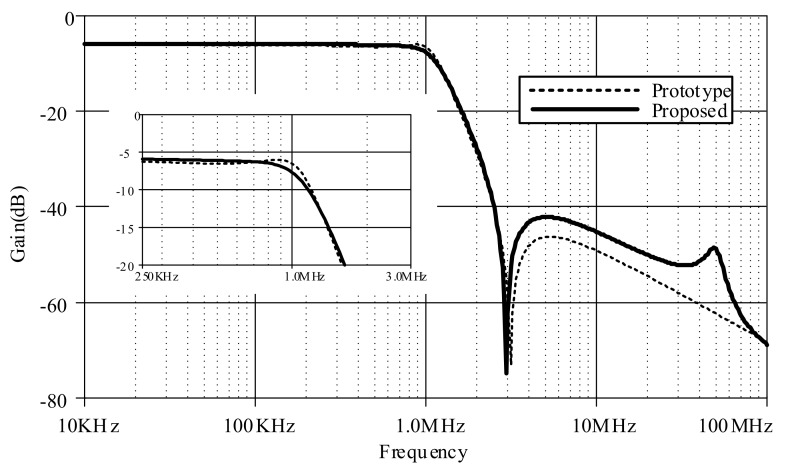
Comparison between magnitude responses of the proposed high-order elliptic ladder LPF and the RLC LPF prototype.

Figure 20 compares between the magnitude responses of the proposed log-domain elliptic ladder LPF and the RLC LPF prototype, given 1 MHz cut-off frequency. In order to achieve the same magnitude response at 1 MHz, *C*_1_ = *C*_3_ = 790 pF, ${C^{\prime }}_{2}$ = 860 pF, *C*_4_ = 50 pF, and *I_B_* = 105 µA for the proposed elliptic LPF (Figure 12); and *R_S_* = *R_L_* = 1Ω, *C*_1_ = *C*_3_ = 158 nF, *L*_2_ = 172 nH, and *C*_4_ = 9 nF for the RLC LPF prototype (Figure 1). The magnitude responses are of similar shape despite small differences in pass band ripple and stopband attenuation.

**Figure 21:**
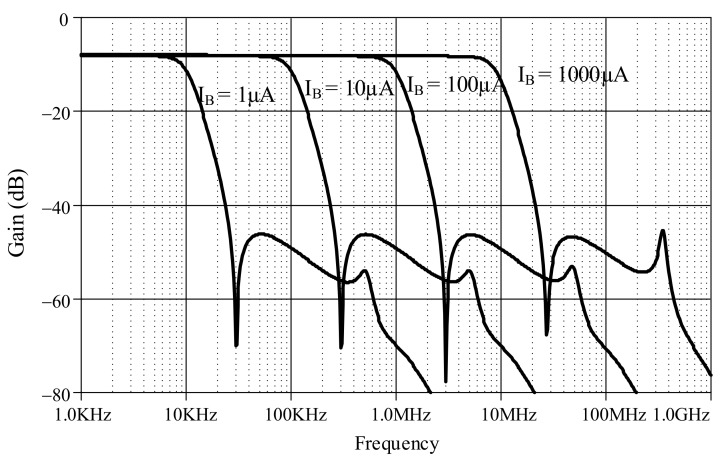
Magnitude responses of the proposed high-order elliptic ladder LPF under varying *I_B_*.

Figure 21 shows the magnitude responses of the high-order elliptic ladder LPF under varying *I_B_* (i.e., 1 µA, 10 µA, 100 µA, and 1000 µA), given *C*_1_ = *C*_3_ = 790 pF, ${C^{\prime }}_{2}$ = 860 pF, *C*_4_ = 50 pF (Figure 12). This simplified the proposed elliptic LPF structure, compared with [21] which required two substantially diverse current gains. The accuracy performance of the proposed LPF is more agreeable with theory, in comparison with the conventional complex high-order elliptic LPF.

**Figure 22:**
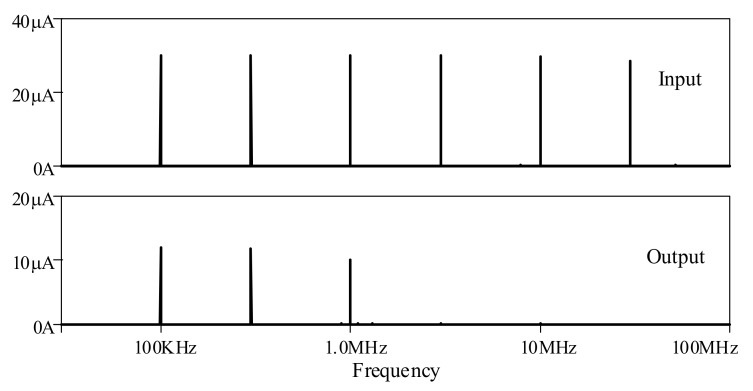
Multi-tone testing of the proposed high-order elliptic ladder LPF.

Multi-tone testing was carried out with the proposed elliptic ladder LPF by biasing input between 100 kHz, 300 kHz, 1 MHz, 3 MHz, 10 MHz, and 30 MHz, given *I_B_* of 120 μA for 1MHz passband. Figure 22 depicts the frequency spectra of input and output of the elliptic ladder LPF. The LPF output was 1 MHz and below, while the rest with higher frequencies were completely filtered out.

**Figure 23:**
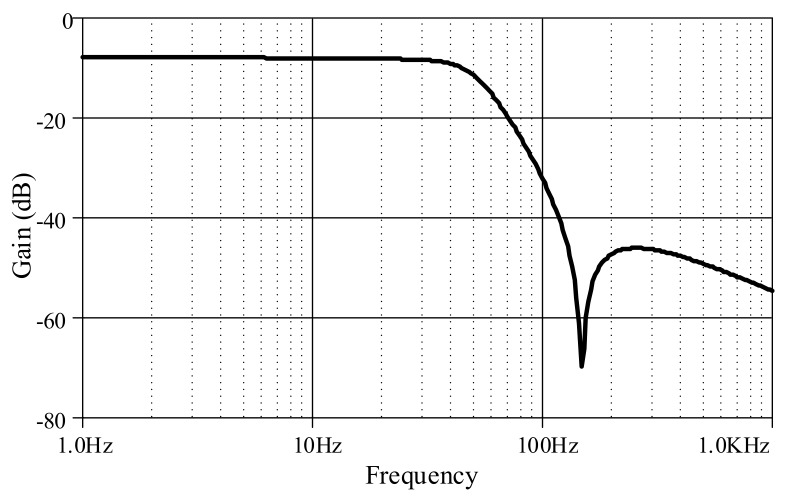
Frequency response of proposed high-order elliptic ladder LPF for biosensor signal.

**Figure 24:**
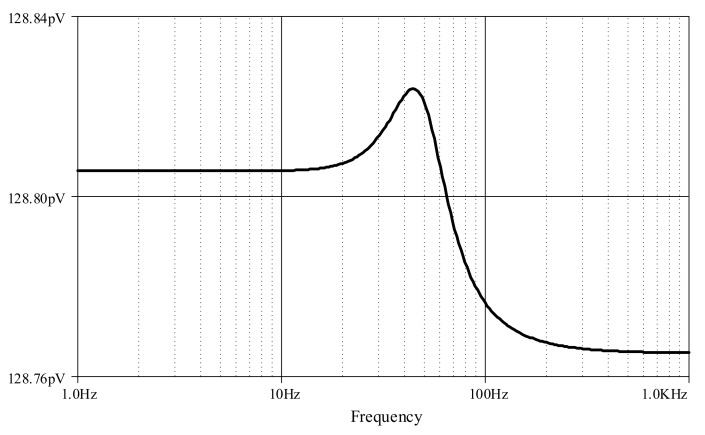
Noise output analysis of proposed high-order elliptic ladder LPF.

**Figure 25:**
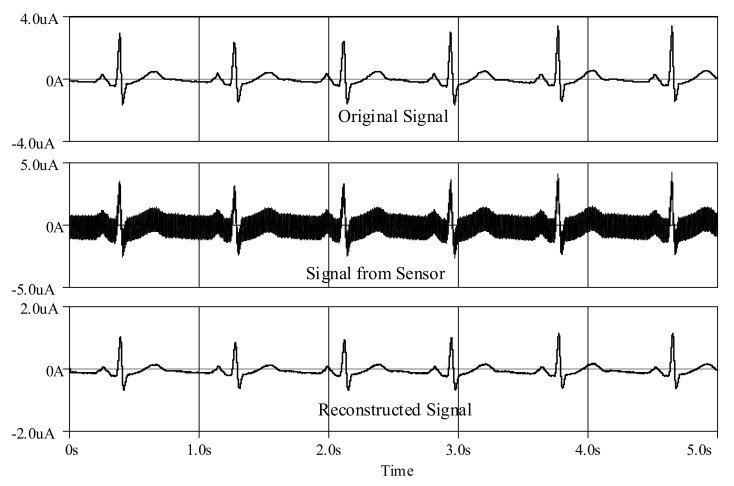
Reconstructed electrocardiogram (ECG) signal using proposed high-order elliptic ladder LPF.

Furthermore, an ECG signal (biosensor signal) was applied to the proposed high-order LPF with certain reconfigurations. Given *C*_1_ = *C*_3_ = 790 nF, ${C^{\prime }}_{2}$ = 860 nF, *C*_4_ = 50 nF with bias current *I* = 5 μA, the frequency response is obtained around 60Hz (Figure 23), with a very small noise output of around 128 pV along the frequency of 1 Hz–1 kHz (Figure 24). In Figure 25, the original ECG signal with high-frequency (random) noises is applied to the proposed LPF filter, and the reconstructed ECG signal is realized without noise as the noise signals at high frequency are removed.

**Figure 26:**
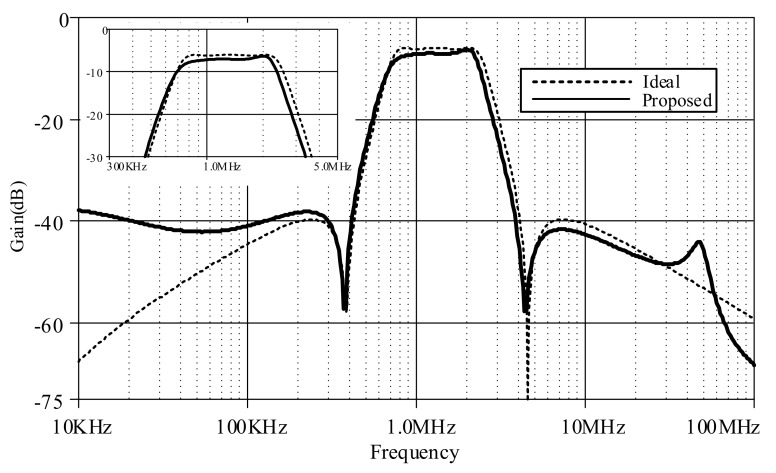
Comparison between magnitude responses of the proposed high-order elliptic ladder BPF and the RLC BPF prototype.

Figure 26 compares the magnitude response of the proposed log-domain elliptic ladder BPF with that of RLC ladder prototype, given 1 MHz cut-off frequency. To achieve the same frequency response at 1 MHz, *C*_1_ = ${C^{\prime }}_{1}$ = *C*_2_ = ${C^{\prime }}_{2}$ = *C*_3_ = ${C^{\prime }}_{3}$ = 500 pF, ${C^{\prime }}_{4}$ = 5 nF, *C*_4_ = 50 pF, and *I_B_* = 105 µA for the proposed high-order elliptic ladder BPF (Figure 15); and *R_S_* = *R_L_* = 1Ω, *C*_1_ = *C*_2_ = *C*_3_ = 120 nF, *L*_1_ = *L*_2_ = *L*_3_ = 120 nH, *C*_4_ = 12 nF, and *L*_4_ = 1.2 µH for the RLC ladder prototype (Figure 3). The magnitude responses are of similar shape with very small differences in pass band ripple and stopband attenuation.

**Figure 27:**
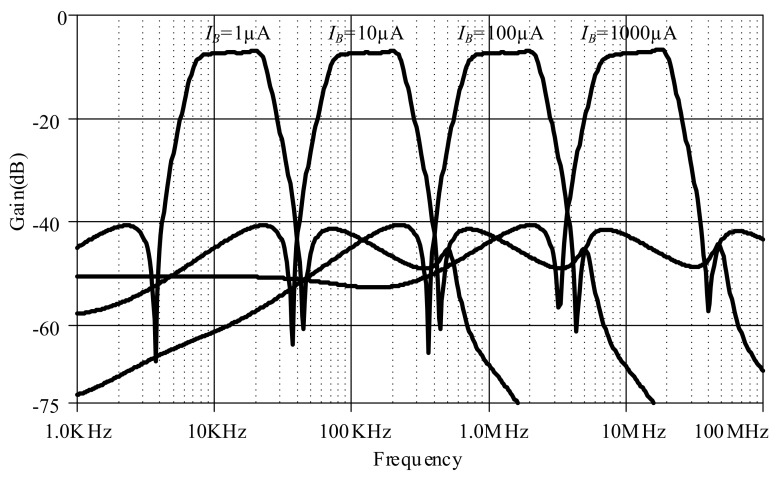
Magnitude responses of the proposed high-order elliptic ladder BPF under varying *I_B._*

Figure 27 shows the magnitude responses of the proposed high-order elliptic ladder BPF under varying *I_B_* (1 µA, 10 µA, 100 µA and 1000 µA), given *C*_1_ = ${C^{\prime }}_{1}$ = *C*_2_ = ${C^{\prime }}_{2}$ = *C*_3_ = ${C^{\prime }}_{3}$ = 500 pF, ${C^{\prime }}_{4}$ = 5 nF, *C*_4_ = 50 pF (Figure 15). This simplified the proposed elliptic BPF structure, compared with [22] which required two substantially diverse current gains. The accuracy performance of the proposed BPF is more agreeable with theory than the conventional elliptic BPF. 

**Figure 28:**
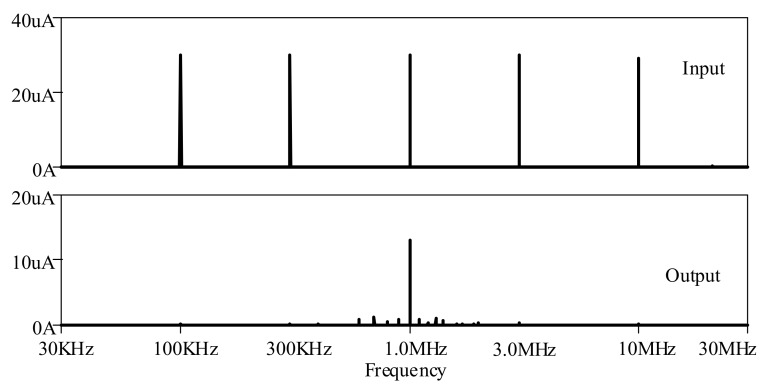
Multi-tone testing of the proposed high-order elliptic ladder BPF.

Multi-tone testing was carried out with the proposed elliptic ladder BPF by biasing input between 100 kHz, 300 kHz, 1 MHz, 3 MHz, 10 MHz, and 30 MHz, given *I_B_* of 80 μA for 1 MHz center frequency. Figure 28 depicts the frequency spectra of input and output of the proposed BP filter. The BPF output was 1 MHz while other frequencies were filtered out.

**Figure 29:**
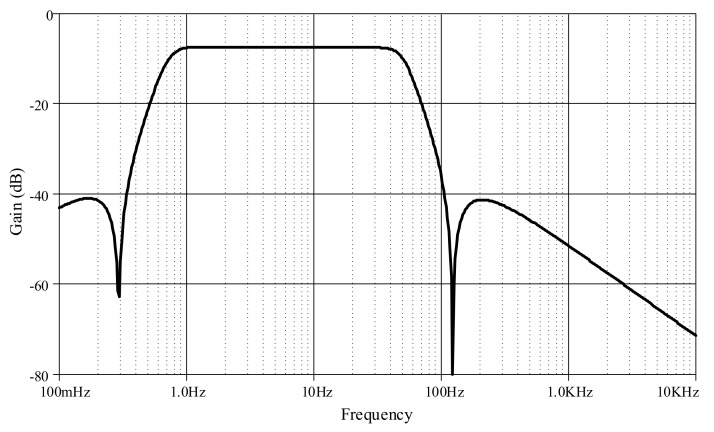
Frequency response of proposed high-order elliptic ladder BPF for biosensor signal.

**Figure 30:**
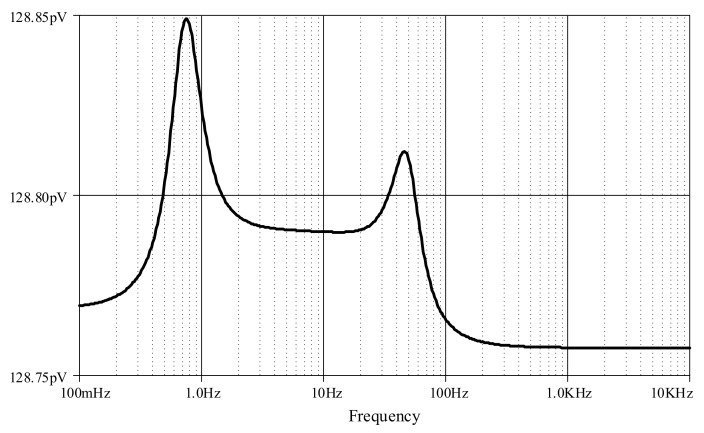
Noise output analysis of proposed high-order elliptic ladder BPF.

**Figure 31:**
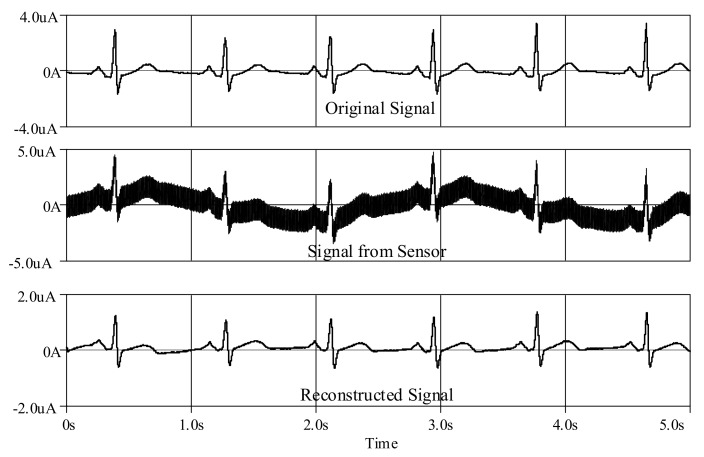
Reconstructed ECG signal using proposed high-order elliptic ladder BPF.

Besides, an ECG signal was applied to the proposed high-order BPF with certain reconfigurations. Given *C*_1_ = *C*_3_ = ${C^{\prime }}_{2}$ = 500 nF, ${C^{\prime }}_{1}$ = *C*_2_ = ${C^{\prime }}_{3}$ = 20 μF, *C*_4_ = 50 nF and ${C^{\prime }}_{4}$ = 200 μF with bias current *I* = 3 μA, the lower and higher cut-off frequencies are, respectively, obtained around 0.6 Hz and 60 Hz, as shown in Figure 29, with a very small noise output of around 128 pV along the frequency of 0.1 Hz–10 kHz (Figure 30). In Figure 31, the original ECG signal with low- and high-frequency (random) noises is applied to the proposed BPF filter, and the reconstructed noiseless ECG signal is achievable as the low- and high-frequency noises in the stop-band of the proposed BPF are completely removed.

## 6. Conclusions

To tackle complexity inherent in conventional elliptic ladder filters, this research presented simple-structure log-domain high-order elliptic ladder LPF and BPF using a lossless differentiator and lossless and lossy integrators. The simplified SFGs of the high-order elliptic ladder LPF and BPF were derived based on RLC prototypes. The elliptic LPF and BPF inherit the tunability, low voltage, and wide dynamic range features of the log-domain principle. The performance of the log-domain elliptic LPF and BPF are in good agreement with the theory. The non-ideality analysis indicated that the smallest and largest capacitor values were 50 pF and 800 pF, resulting in the maximum frequency response of the log-domain high-order elliptic LPF and BPF of around 10 MHz. The proposed log-domain elliptic LPF and BPF can readily be used in biosensor applications by appropriately changing the capacitors. The reconstructed ECG signal has also been verified as having a good performance in terms of the proposed elliptic LPF and BPF. Finally, the power dissipations of the proposed LPF and BPF for biosensor applications consumed around 337 mW and 380 mW, respectively.

## Figures and Tables

**Table 1:** RLC network transformation.

LP Prototype		Transformed BP